# Aurantoside K, a New Antifungal Tetramic Acid Glycoside from a Fijian Marine Sponge of the Genus *Melophlus*

**DOI:** 10.3390/md10010200

**Published:** 2012-01-18

**Authors:** Rohitesh Kumar, Ramesh Subramani, Klaus-D. Feussner, William Aalbersberg

**Affiliations:** Centre for Drug Discovery and Conservation, Institute of Applied Sciences, The University of the South Pacific, Suva, Fiji Islands; Email: kumar_rh@usp.ac.fj (R.K.); feussner_k@usp.ac.fj (K.-D.F.); aalbersberg@usp.ac.fj (W.A.)

**Keywords:** marine sponges, aurantosides, antifungal, tetramic acid glycoside, *Melophlus* sp.

## Abstract

A new tetramic acid glycoside, aurantoside K, was isolated from a marine sponge belonging to the genus *Melophlus.* The structure of the compound was elucidated on the basis of spectroscopic analysis (^1^H NMR, ^1^H–^1^H COSY, HSQC, and HMBC, as well as high-resolution ESILCMS). Aurantoside K did not show any significant activity in antimalarial, antibacterial, or HCT-116 cytotoxicity assays, but exhibited a wide spectrum of antifungal activity against wild type *Candida albicans*, amphotericin-resistant *C. albicans*, *Cryptococcus neoformans*, *Aspergillus niger*, *Penicillium* sp., *Rhizopus sporangia* and *Sordaria* sp.

## 1. Introduction

Sponges (Porifera) are multicellular organisms that remain the most important source in the field of marine drug discovery. Sponges are a well-known source of new/novel bioactive natural products of pharmaceutical and medical relevance [1,2,3,4,5,6,7]. Marine sponges belonging to the genus *Melophlus* (Astrophorida, Ancorinidae) are promising sources of structurally unusual and diverse secondary metabolites. *Meophlus* spp. sponges have been reported to produce compounds primarily from three classes, specifically tetramic acid derivatives [8,9,10], saponin derivatives [11,12], and depsipeptides [13] with potent and varied biological activities. The melophlins [8,9,10] are tetramic acids with long-chain saturated alkane side chains and differ significantly from the aurantosides, another sponge-produced class of tetramic acids containing chlorinated polyene side chains and displaying di- and tri-saccharide substituents attached to nitrogen. Aurantosides have been reported to display antifungal and cytotoxic activities and have been isolated from sponges of the family Theonellidae [14,15,16,17,18] but to date have not been reported from *Melophlus*. As part of our continuing search for new bioactive metabolites from Fijian invertebrates, we investigated an extract from *Melophlus* sp. exhibiting significant antifungal activity. We report here the isolation, structure elucidation, and biological activity of a novel compound aurantoside K (**1**) resulting from these studies.

## 2. Results and Discussion

**Chart 1:**
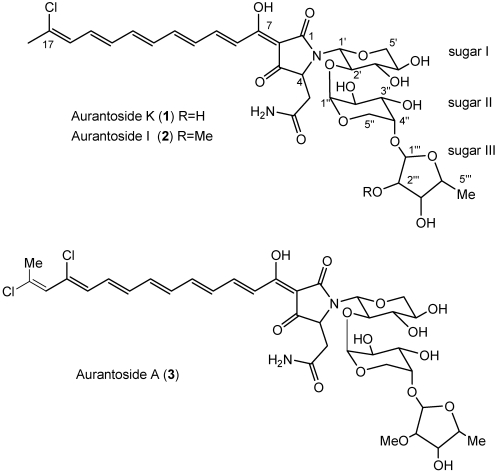
Compound **1** and the known aurantosides A and I.

The crude MeOH extract was separated by solvent partitioning between 90% MeOH_(aq)_ and hexane. The MeOH fraction was concentrated under vacuum and fractionated by flash chromatography on ODS-A to afford ten fractions, of which three were toxic to both wild type *C. albicans* and amphotericin-resistant *C. albicans*, at 250 µg/disc. The +HRESILCMS profile for the active fractions displayed mass spectral isotopic patterens suggestive of the presence of one or two chlorine atoms for each of the three major compounds. The mass spectral data were used to search the MarinLit database (2011, vpc 15.5) [19], allowing the identification of aurantosides I [17] (**2**) and A [14] (**3**) for two of the three active compounds. Compound **1** contained one chlorine atom and did not match any of the candidate formulas from MarinLit. Among the aurantosides (**1–3**) detected in this study (Chart 1), compound **1** showed unknown molecular mass and was subjected to further purification by reversed-phase HPLC to afford the new tetramic acid glycoside aurantoside K (**1**). The compound was isolated as a bright orange amorphous solid. 

**Figure 1:**
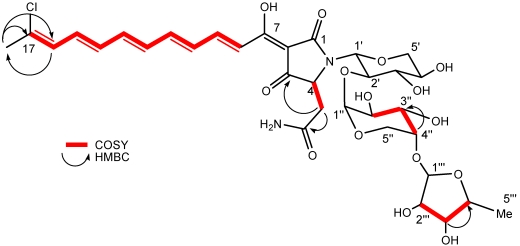
The key ^1^H–^1^H COSY and HMBC correlations of compound **1**.

The +HRESILCMS analysis of aurantoside K (**1**) supported a molecular formula of C_33_H_44_ClN_2_O_15_ (*m*/*z* 743.2428 [M + H]^+^); calcd. for C_33_H_44_ClN_2_O_15_, 743.2425; ∆ = 0.4 ppm, (−ESILCMS *m/z* 741.43 [M − H]^−^), which was consistent with the NMR data. The ^1^H NMR spectra (Table 1) of compound **1** was nearly superimposable on aurantoside I [17], except for the absence of the C-2"' methoxy group on the sugar unit, which is replaced by a hydroxyl group by interpretation of 1D and 2D NMR spectra (Figure 1). This was also confirmed by the ESILCMS fragmentation patterns of **1** and **2**. The +HRESILCMS disclosed that compound **1** was 14 amu (CH_2_ unit) lighter than aurantoside I (**2**) further affirming the replacement of a methoxy group with a hydroxyl group. Interpretation of the COSY spectrum revealed that **1** had a polyene chain from C-8 to C-16 with *E* configurations. Two carbonyl-attached methylene (H-5a and H-5b, 2.54 and 2.59 ppm) were observed in DMSO-*d*_6_ which showed HSQC correlations to C-5 at 36.3 ppm indicating that the aliphatic methylene protons are attached to C-5. These protons (H-5) also showed HMBC correlations to a quaternary carbonyl carbon and the amide carbonyl carbon suggesting that C-3, C-4, C-5 and C-6 are part of the tetramic acid moiety or attached to it. The COSY correlations of proton H-4 and the aliphatic methylene H-5 also indicate the same tetramic acid core found in aurantoside I (**2**). The chlorine atom was placed on C-17 based on its ^13^C chemical shift (δ_C_ 133.0 ppm) and the splitting pattern of the neighboring proton H-16 (δ_H_ 6.44 ppm, d, *J* = 10.2 Hz) and H_3_-18 (δ_H_ 2.22 ppm, s) (Figure 1). 

**Table 1:** NMR Spectroscopic Data (500/125 MHz, DMSO-*d*_6_) for Aurantoside K (**1**).

Position	δ_H_ (*J* in Hz)	δ_C_*^a^*	COSY	HMBC *^b^*
1	-	*c*		
2	-	*c*		
3	-	170.3		
4	4.16 br	63.8	5a, 5b	
5a	2.54, m	36.3	4	3, 6
5b	2.59, m		4	3, 6
6	-	170.3		
7	-	*c*		
8	6.97 br, d (18.3)	143.6	9	
9	6.61, dd (10.4, 16.9)	130.6	8, 10	
10	6.38, dd (11.3, 13.2)	127.5	9, 11	
11	7.97, dd (13.6, 13.8)	138.4	10, 12	
12	7.12, dd (12.4, 13.1)	120.2	11, 13	
13	7.55, dd (15.1, 11.1)	144.2	12, 14	
14	6.71, d (11.8)	130.9	12, 13, 15	
15	6.58–6.48, m	133.4	14	
16	6.44, d (10.2)	125.6	18	18
17	-	133.0		
18	2.22, s	26.0	16	16, 17
1'	5.21, m	79.0		
2'	4.37 br	79.5		
3'	4.36 br	83.8		
4'	3.53, m	63.66		
5a'	3.27, d (8.8)	77.3		2'
5b'	3.39, dd (12.3, 6.6)			2'
1"	4.79, s	102.3		
2"	3.59, m	68.4	4"	
3"	3.50, m	68.9	4"	
4"	3.18, d (11.6)	63.67	2", 3"	1", 3"
5"	4.24, d (11.6)	74.1		
1"'	4.93, m	102.5		
2"'	3.08, t (10.9)	67.57	3"'	
3"'	3.71, dd (11.2, 5.5)	67.60	2"', 4"'	4"'
4"'	3.38, m	68.5	3"'	
5"'	1.22 *^d^*	*c*		

*^a^* obtained from 2D NMR; *^b^* HMBC correlations are from proton(s) stated to the indicated carbon; *^c^* Not observed; *^d^* obtained from COSY spectrum.

The reported mass spectrum of aurantoside I [17] displayed a pseudomolecular ion at *m/z* 757 ([M + H]^+^) and a fragment at *m/z* 627 corresponding to loss of 130 Da that was assigned to loss of 5-deoxy-2-*O*-methylarabinofuranose. Compound **1** (*m/z* = 743 [M + H]^+^) also showed a distinct fragment ion at *m/z* 627 however, the difference was 116 daltons, further indicating that the sugar unit which is cleaved off is indeed 14 amu smaller than the sugar III of aurantoside I (**2**). Moreover, the psuedomolecular ion of compound **1** at *m/z* 743 produced prominent fragment ions at *m/z* 627 [M + H − sugar III]^+^, 495 [M + H − sugar II + III]^+^ and at 363 [M + H − sugar I + sugar II + III]^+^, which were generated due to the cleavage of the three glycosidic bonds, confirming the trisaccharide series. Although the ^1^H NMR signals were complicated by broad and overlapping signals, two distinct anomeric protons were identified from the ^1^H–^1^H COSY spectrum (Figure 1). The high field signal (δ_H_ 4.79 ppm) was assigned to H-1" of the arabinopyranose structure (sugar II) while the other anomeric proton H-1"', (δ_H_ 4.93 ppm) was assigned to the 2"'-des-*O*-methyl derivative of 5-deoxypentofuranose unit (sugar III) with the signal at δ_H_ 1.22 ppm due to the methyl group of sugar III. Absence of an *O*-methyl signal in the NMR spectra and an upfield shift of C-2"' (δ_C_ 67.6 ppm) were consistent with replacement of the 2"' OMe group by a hydroxyl group when compared to the known aurantosides [14,17]. The remainder of the structure of **1** was confirmed by comparison of the 1D and 2D NMR data against those reported for the other known aurantosides, confirming the flat structure of **1** as a new congener designated as aurantoside K. 

The biosynthetic relationship between aurantoic acid and aurantosides has not been established to date. However, according to the literature [14,15,16,17,20], the marine-derived tetramic acid glycosides aurantosides A–F and rubrosides A–H are all derived from L-aspartic acid and carry D-saccharides which may well be indicative of a common biosynthetic pathway to these polyene tetramic acid metabolites. Furthermore, aurantosides are homogeneous in terms of the polyene chain, however the sugar moiety especially trisaccharides plays an important role for the biological activity.

The biological activities of the purified compound **1** was examined in cytotoxicity [21], antimalarial [22,23,24], antibacterial [25] and antifungal [9,25] bioassays. Aurantoside K (**1**) failed to show any significant activity against HCT-116 human cancer cell lines and it did not exhibit antibacterial activity against methicillin-resistant *Staphylococcus aureus* or vancomycin-resistant *Enterococcus faecium.* No antimalarial activity against *Plasmodium falciparum* parasites was shown by compound **1**. However, **1** displayed appreciable antifungal activity against amphotericin-resistant *C. albicans* and wild type *C. albicans* with the minimum inhibitory concentration (MIC) at 31.25 μg/mL and 1.95 μg/mL, respectively. Furthermore, **1** displayed potent inhibitory activity against the pathogenic yeast *Cryptococcus neoformans* with a clear inhibition zone of 14 mm diameter at the concentration of 100 μg/disc. Additionally, **1** significantly inhibit the pathogenic fungi *A. niger* (28 mm), *Penicillium* sp. (31 mm), *R. sporangia* (21 mm) and *Sordaria* sp. (29 mm) at a test concentration of 100 μg/disc. 

## 3. Experimental Section

### 3.1. General Experimental Procedure

Optical rotation was measured on a Rudolph automatic polarimeter. UV spectrum was acquired in spectroscopy grade methanol using a PerkinElmer *Lambda 35* spectrophotometer. NMR spectra (1D and 2D NMR; COSY, HSQC, and HMBC) were acquired on a UXNMR, Bruker Analytische Messtechnik GmbH NMR spectrometer using a 5 mm broadband probe, and were referenced to the residual solvent (δ_H_ 2.50 ppm and δ_C_ 39.5 ppm for DMSO-*d*_6_) operating at 500/125 MHz. Electrospray ionization mass spectra (ESIMS) were acquired using an Agilent 1100 Series separations module equipped with an Agilent 1100 Series LC/MSD mass detector in both positive and negative ion modes. Purification was done on a Zorbax ODS 5 µm 4.6 × 250 mm column. Analytical and semi-preparative HPLC was performed using a Waters 515 pump connected to a 2487 UV-Vis detector. TLC analyses were carried out using glass plate pre-coated silica gel 60 RP-18 F_254_S (Merck, Darmstadt, Germany). Analytical grade solvents were utilized for chromatographic analysis. Burdick and Jackson high purity solvents were used for HPLC while Riedel-de Haen, Chromasolv LCMS grade solvents were used for LCMS.

### 3.2. Biological Material

The sponge *Melophlus* sp. was collected by hand using SCUBA at a depth of 10 m from Cicia, Lau group, Fiji Islands (17°47'33''S, 179°23'94''W) in September 2008 during a three week biodiversity expedition in the Central Lau group. The sponge (brownish maroon external color and dark green internal color) was soft to touch and consisted of a few 5–9 mm diameter oscules. This sponge produced a bright orange secretion which would stain any surface it came into contact with, suggesting the presence of interesting chemistry. The sponge was identified by Prof. John Hooper, Queensland Museum, Australia. A voucher specimen (G-0634) has been preserved at the Marine Reference Collection, The University of the South Pacific, Fiji Islands and at Georgia Institute of Technology, USA. 

### 3.3. Extraction and Isolation

The frozen sponge (1220 g wet weight) was extracted with MeOH at room temperature, yielding 15 g of crude extract after solvent removal *in vacuo*. A portion of the crude extract (6.5 g) was partitioned between 90% MeOH_(aq)_ and hexane. The MeOH extract was purified by flash chromatography on ODS-A (6 nm S–150 µm) using a stepwise gradient solvent system of H_2_O-MeOH to afford ten fractions (F1–F10). Fractions F7–F9 (eluted with 70% to 90% MeOH_(aq)_) were pooled due to their significant anti-*Candida* activity. These fractions contained chlorinated compounds as illustrated by +HRESILCMS. The combined fraction (F7–F9) was further purified by semi-preparative RP-HPLC (Zorbax ODS 5 µm 4.6 × 250 mm column) with 45% CH_3_CN_(aq)_ and 0.1% TFA followed by 40% CH_3_CN_(aq)_ and 0.1% TFA at a flow rate of 3 mL/min and was detected at 254 nm to furnish pure aurantoside K (**1**, 5 mg).

*Aurantoside K* (**1**):orange solid, [α]^27^_D_ −127° (*c* 0.2, MeOH): UV (MeOH) λ_max_ (log ε) 434 (3.95), 265 (2.41) nm; NMR data, see Table 1; HRESIMS *m/z* 743.2428 [M + H]^+^ (calcd. for C_33_H_44_ClN_2_O_15, _743.2425).

### 3.4. Agar Diffusion Assay

Susceptibility discs (6 mm in diameter) were impregnated with 100 µg of the pure compound dissolved in MeOH and placed on potato dextrose agar (PDA) plates inoculated with the test fungal pathogens *A. niger*, *Penicillium* sp., *R. sporangia* and *Sordaria* sp. The plates were observed for zones of inhibition after 7–10 days of incubation at room temperature. The compound was also assayed against *Cr. neoformans* ATCC 32045 which was inoculated on PDA agar plates, and zones of inhibition were recorded after 24 h of incubation at 27 °C. In all cases, for the controls containing only the respective amount of solvent, no growth inhibition was observed. The procedure was the same as previously reported [9].

### 3.5. Proliferation Assay

Antimalarial activity was determined with a SYBR Green based parasite proliferation assay, adapted from Smilkstein [22] and Bennett [23]. Briefly, *P. falciparum* parasites (3D7 strain MR4/ATCC, Manassas, VA) were cultured in human O+ erythrocytes as previously described [24]. Compound **1** was diluted in complete medium and 40 µL transferred to 96-well assay plates. To this 80 µL of complete media with 3D7 infected erythrocytes were dispensed in order to obtain a 2.5% hematocrit and 0.5% parasitemia in the assay. Uninfected erythrocytes were dispensed into the background wells at the same final hematocrit. Plates were incubated for 72 h in a low oxygen environment (96% N_2_, 3% CO_2_, 1% O_2_) in a modular incubation chamber. The plates were sealed and placed in a −80 °C freezer overnight then thawed, and 120 µL of lysis buffer (20 mM Tris-HCl, pH 7.5, 5 mM EDTA, 0.08% Triton X-100, 0.008% saponin with 0.2 µL/mL Sybr Green I) was dispensed into each well and incubated at 37 °C in the dark for 6 h. The plates were read with a Molecular Devices SpectraMAX Gemini EM at ex: 495 nm, em: 525 nm with 515 nm cut-off.

### 3.6. MTS Dye Conversion Assay

Compound **1** was evaluated against human colon cancer cell lines, HCT-116. *In vitro* cytotoxicity was tested with the (3-(4,5-dimethylthiazol-2-yl)-5-(3-carboxylmethoxyphenyl)-2-(4-sulfophenyl)-2*H*-tetrazolium inner salt) MTS dye conversion assay as described previously [21].

### 3.7. Minimum Inhibitory Concentration for Antibacterial and Antifungal Activity

The isolated pure compound was dissolved in MeOH at 10 mg/mL concentration and diluted further to give required concentrations (µg/mL) such as 500, 250, 125, 62.5, 31.25, 15.62, 7.8, 3.9, 1.9, 0.97, 0.48, 0.24, 0.12, 0.06, 0.03 and 0.01. The diluted solutions (100 µL) were added to separate wells on a 96 wells plate. An inoculum of 100 µL from 24 h old culture of each test human pathogens, wild type *C. albicans* ATCC 32354 and amphotericin resistant *C. albicans* ATCC 90873, were inoculated separately in each well. The antifungal agent nystatin and the solvent MeOH were included in the bioassay as positive and negative controls, respectively. The treated cultures were incubated for 24 h at 37 °C. Replicates were maintained for each treatment. The MIC was defined as the lowest concentration of the purified compound/nystatin showing no visible growth after the incubation time. In the case of antibacterial assays were performed using methicillin-resistant *S. aureus* (MRSA, ATCC 10537) and vancomycin-resistant *E. faecium* (VREF, ATCC 12952) as test pathogens. Vancomycin and chloramphenicol were used as positive controls for MRSA and VREF, respectively, and MeOH was used as negative control. 

## 4. Conclusions

In conclusion, we described a new tetramic acid glycoside, aurantoside K (**1**) from a marine sponge *Melophlus* sp. Aurantoside K (**1**) was evaluated in different bioassays that displayed promising antifungal activity against a wide spectrum of fungal pathogens. However, it showed no appreciable activity against human cancer cell lines, the malaria parasite *P. falciparum*, or any bacteria tested in our assays.

## Supplementary Information

1D and 2D NMR spectra and HRESI mass spectral data for aurantoside K are available as supplementary information.

## Supplementary Files

Supplementary File 1: PDF-Document (PDF, 409 KB)
