# Supplementary material for: Aurantoside K, a New Antifungal Tetramic Acid Glycoside from a Fijian Marine Sponge of the Genus Melophlus
Source: Mar Drugs. 2012 Jan 18;10(1):200–8. doi: 10.3390/md10010200 (PMC3280539; doi:10.3390/md10010200)

Supplementary Information

Aurantoside K, a New Antifungal Tetramic Acid Glycoside from a Fijian Marine Sponge of the Genus *Melophlus*

Rohitesh Kumar, Ramesh Subramani*, Klaus-D. Feussner and William Aalbersberg

*Corresponding Author; E-Mail: subramani_r@usp.ac.fj

<u>Table of Contents</u>

^{1}H NMR spectrum of Aurantoside K (**1**)

COSY spectrum of Aurantoside K (**1**)

HSQC spectrum of Aurantoside K (**1**)

HMBC spectrum of Aurantoside K (**1**)

+ HRESILSMS spectrum of Aurantoside K (**1**)

^{1}H NMR (500 MHz, DMSO-d_6) spectrum of Aurantoside K (**1**)

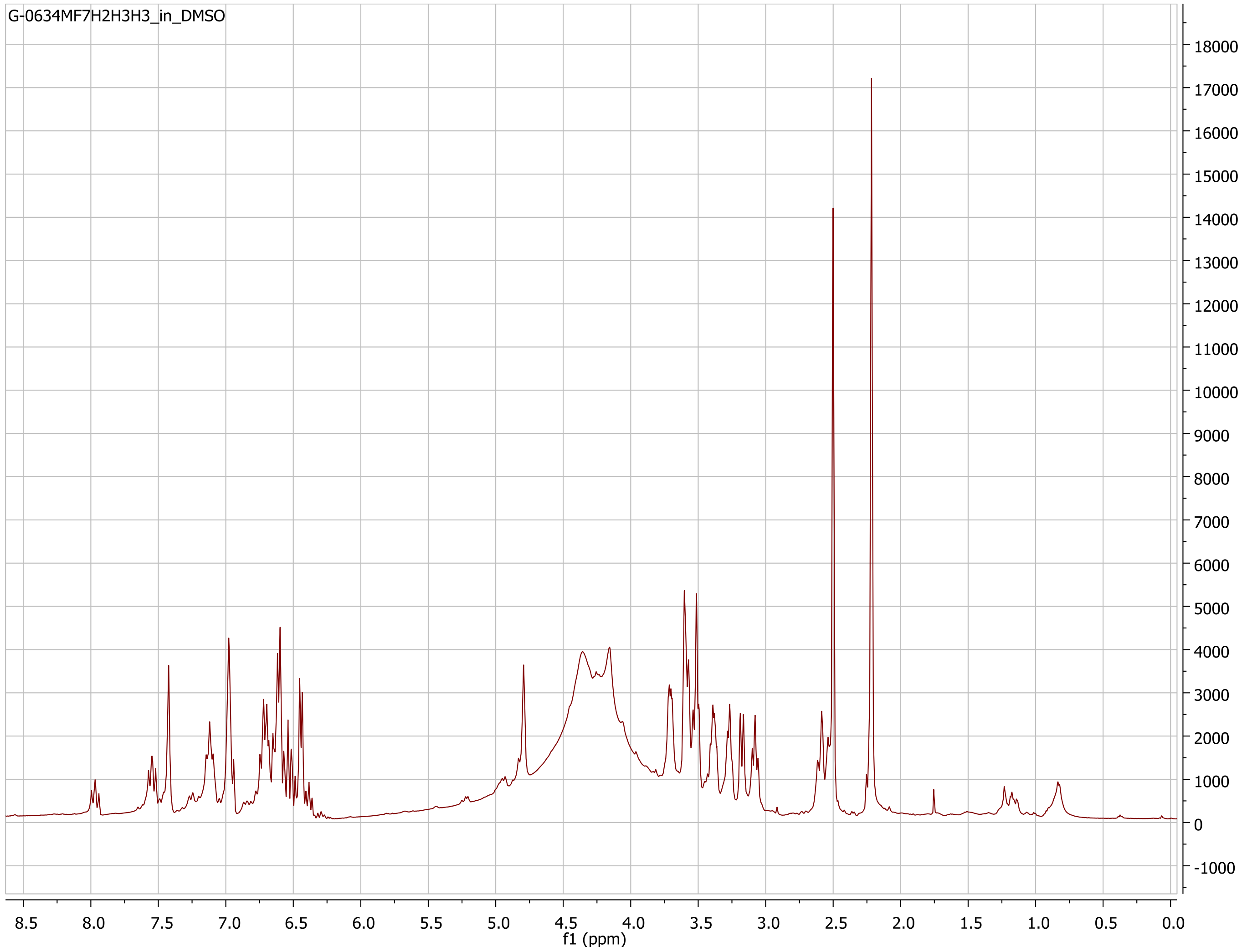

COSY (125 MHz, DMSO-d_6) spectrum of Aurantoside K (**1**)

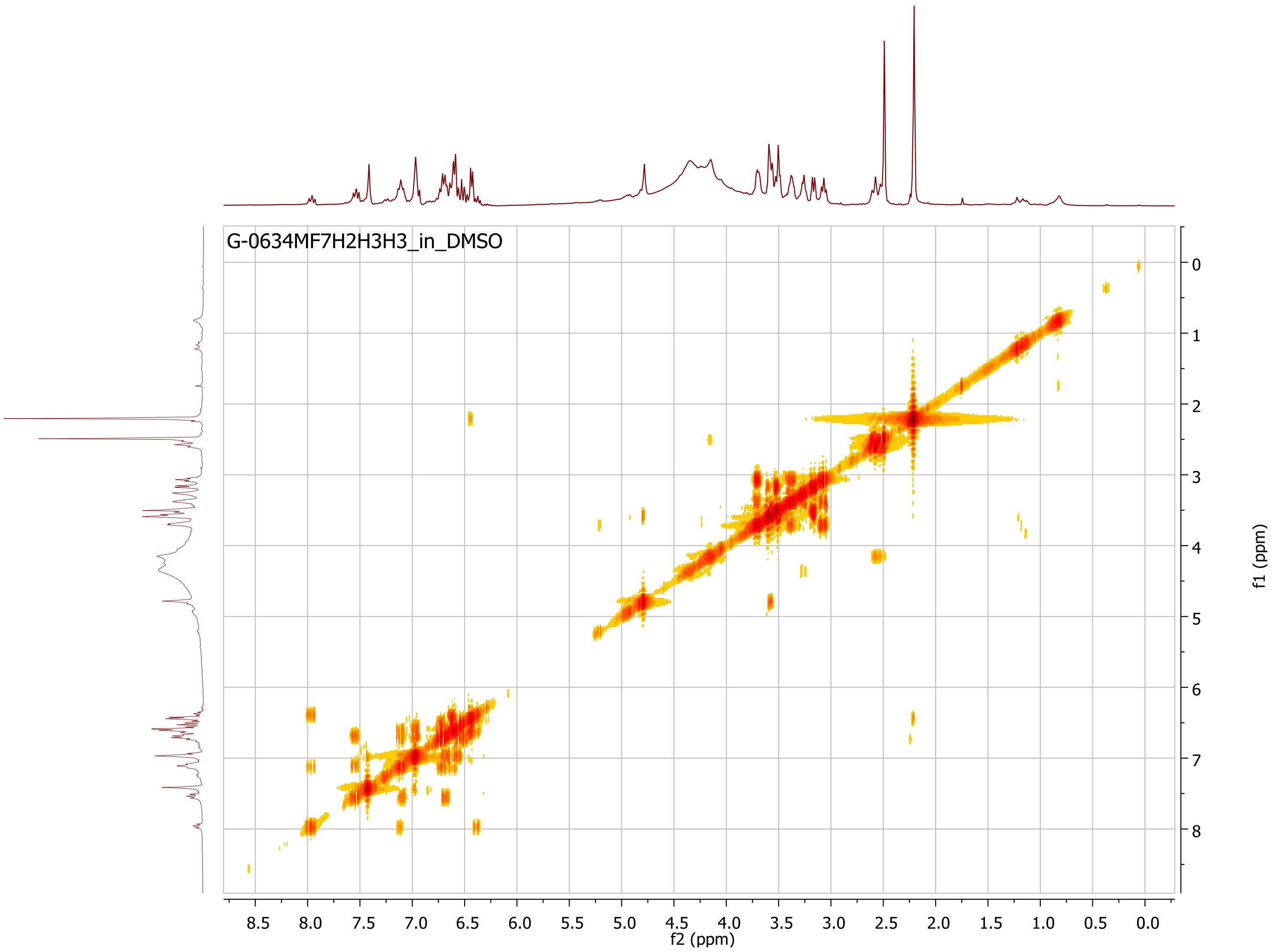

HSQC (125 MHz, DMSO-d_6) spectrum of Aurantoside K (**1**)

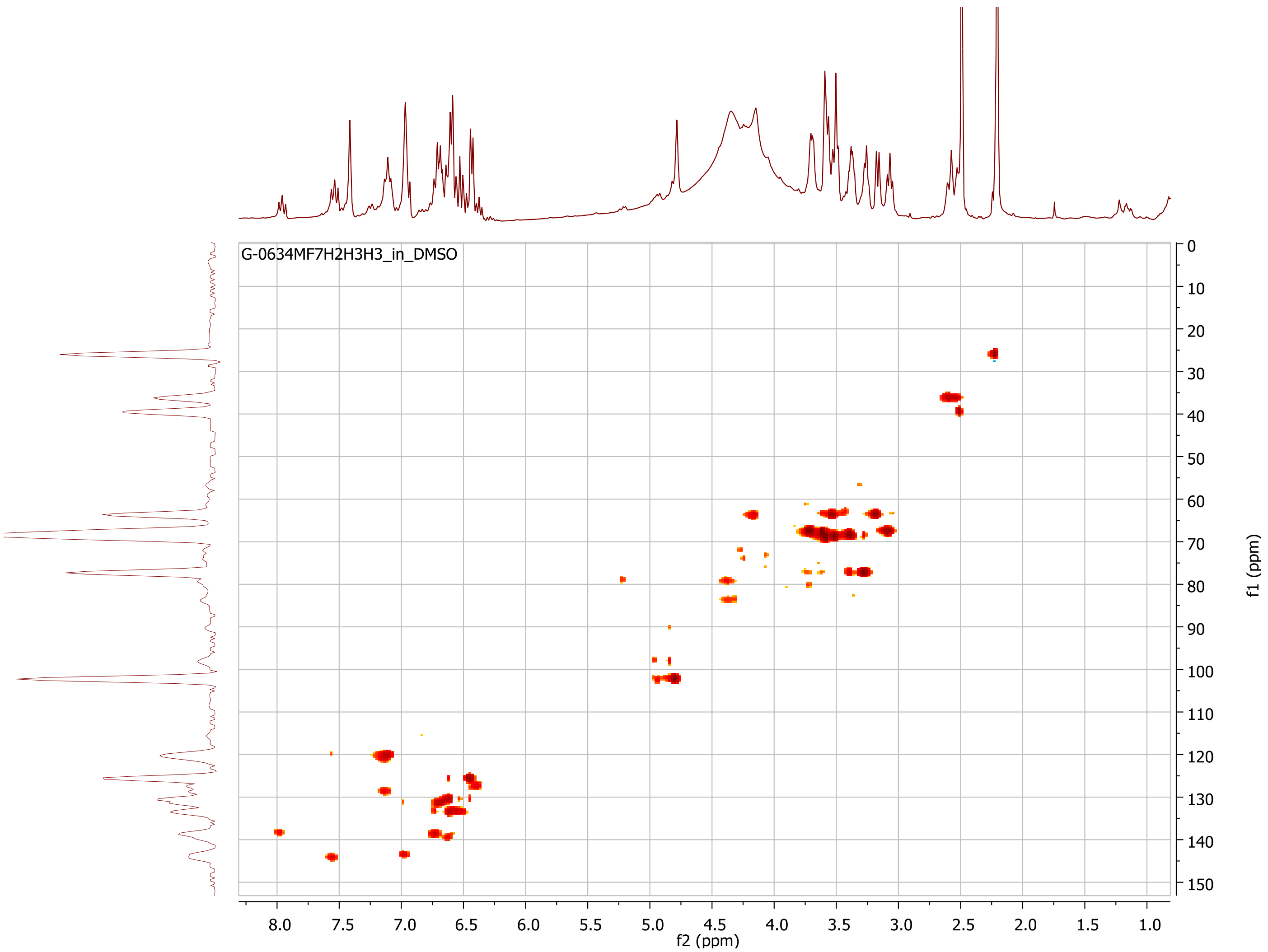

HMBC (125 MHz, DMSO-d_6) spectrum of Aurantoside K (**1**)

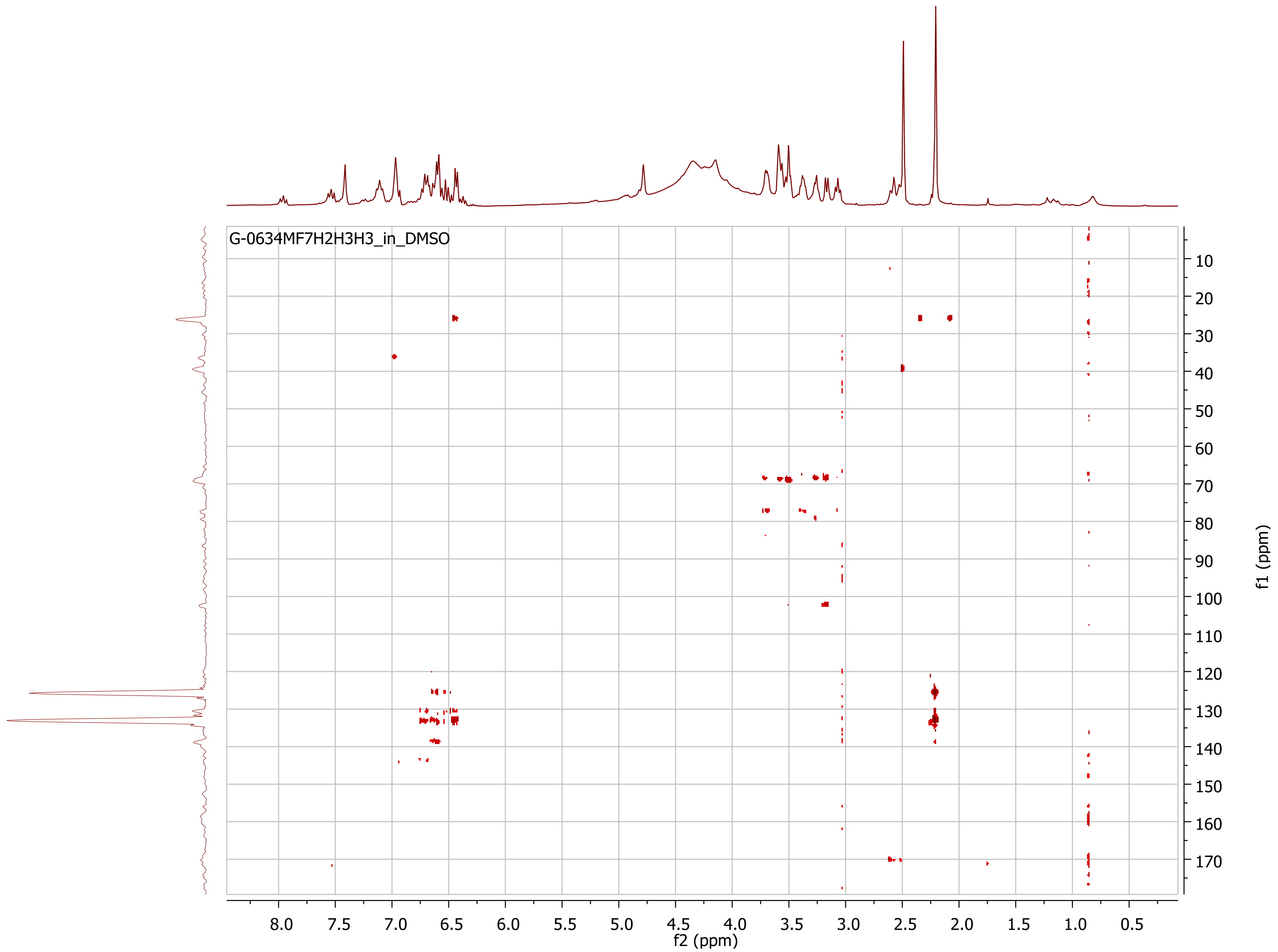

+ HRESILCMS spectrum of Aurantoside K (**1**)

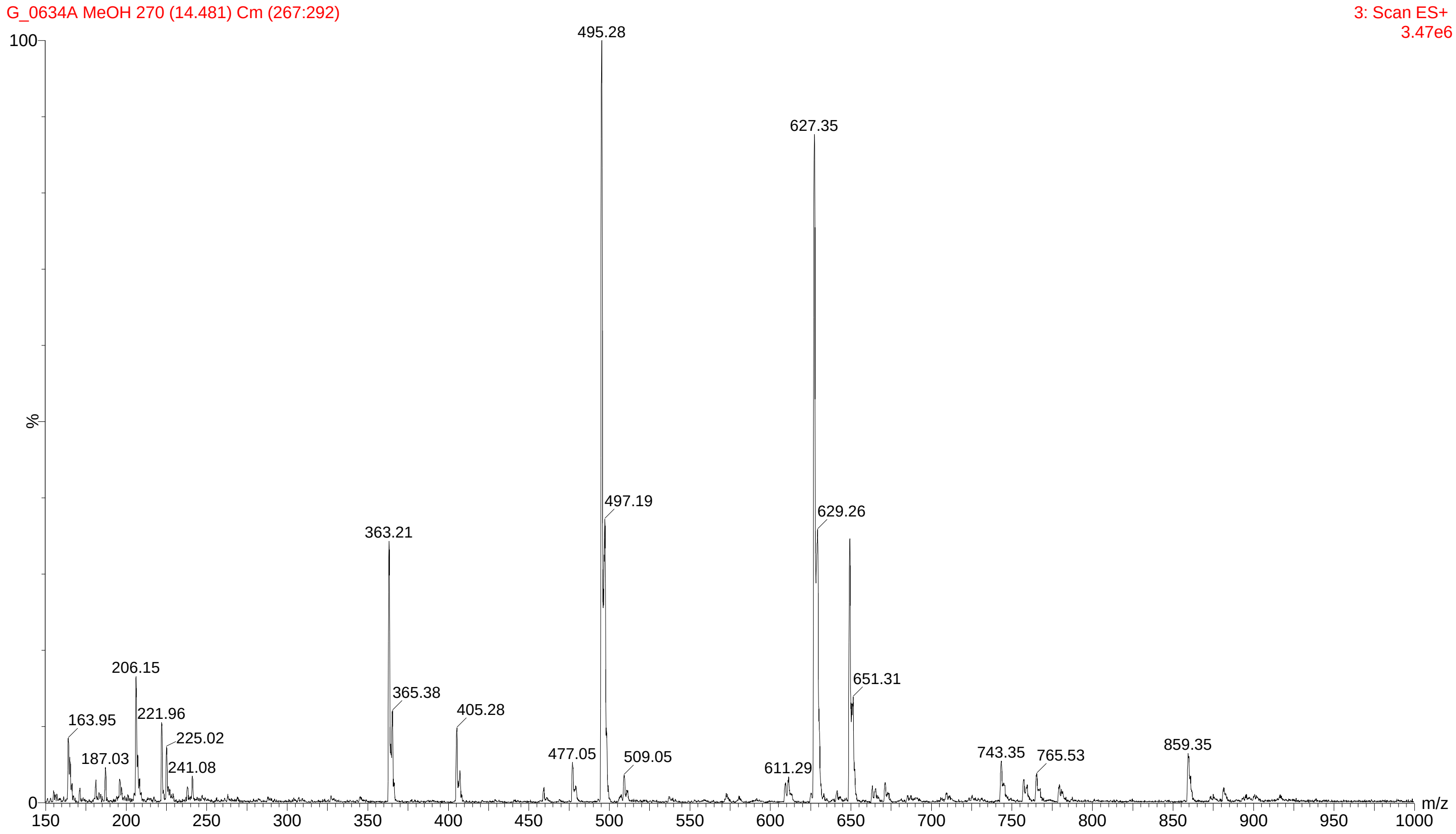

Supplement: Supplementary File 1: — PDF-Document (PDF, 409 KB) [file marinedrugs-10-00200-s001.pdf]
